# Adjuvant chemotherapy of pT1a and pT1b breast carcinoma: results from the NEMESI study

**DOI:** 10.1186/1471-2407-12-158

**Published:** 2012-04-30

**Authors:** Stefania Gori, Matteo Clavarezza, Salvatore Siena, Jennifer Foglietta, Emiliana Tarenzi, Monica Giordano, Annamaria Molino, Claudio Graiff, Vittorio Fusco, Oscar Alabiso, Editta Baldini, Teresa Gamucci, Giuseppe Altavilla, Davide Dondi, Marco Venturini

**Affiliations:** 1 Oncologia Medica, Ospedale Santa Maria della Misericordia, Azienda Ospedaliera di Perugia, Perugia, Italy; 2 Oncologia, Ospedale Sacro Cuore-Don Calabria, Negrar, (VR), Italy; 3 Struttura Complessa di Oncologia Falck, Ospedale Niguarda Ca’ Granda, Milan, Italy; 4 UO Oncologia, Azienda Ospedaliera Sant’Anna, Como, Italy; 5 U.O.C. di Oncologia dell’Ospedale Civile Maggiore, Azienda Ospedaliera-Universitaria di Verona, Verona, Italy; 6 Oncologia Medica, Ospedale di Bolzano, Bolzano, Italy; 7 Oncologia, Azienda Ospedaliera di Alessandria, Alessandria, Italy; 8 SC Oncologia, Azienda Ospedaliero-Universitaria “Maggiore della Carità”, Novara, Italy; 9 Oncologia Medica, Ospedale Campo di Marte, Lucca, Italy; 10 UO Oncologia Medica, Ospedale S.S. Trinità, Sora (FR), Sassari, Italy; 11 Oncologia Medica, Università degli Studi Messina, Messina, Italy; 12 Medical & Scientific Department, Sanofi-Aventis, Milan, Italy; 13 Division of Medical Oncology, Ospedale S. Maria della Misericordia, Azienda Ospedaliera Perugia, via Dottori 1, Perugia, 06122, Italy

**Keywords:** pT1a and pT1b breast cancer, Adjuvant chemotherapy, Adjuvant hormonal therapy

## Abstract

**Background:**

The prognosis of pT1a-pT1b breast cancer (BC) used to be considered very good, with a 10-y RFS of 90%. However, some retrospective studies reported a 10-y RFS of 81%–86% and suggested benefit from adjuvant systemic therapy.

**Methods:**

To evaluate the variables that determined the choice of adjuvant chemotherapy and the type of chemotherapy delivered in pT1a-pT1b BC, we analysed the small tumours enrolled in the NEMESI study.

**Results:**

Out of 1,894 patients with pathological stage I-II BC enrolled in NEMESI, 402 (21.2%) were pT1a-pT1b. Adjuvant chemotherapy was delivered in 127/402 (31.59%). Younger age, grading G3, high proliferative index, ER-negative and HER2-positive status were significantly associated with the decision to administer adjuvant chemotherapy. An anthracycline without taxane regimen was administered in 59.1% of patients, anthracycline with taxane in 24.4%, a CMF-like regimen in 14.2% and taxane in 2.4%. Adjuvant chemotherapy was administered in 88.4% triple-negative and 73.46% HER2-positive pT1a-pT1b BC. Adjuvant trastuzumab was delivered in 30/49 HER2-positive BC (61.2%).

**Conclusions:**

Adjuvant chemotherapy was delivered in 31.59% T1a-pT1b BC treated at 63 Italian oncological centres from January 2008 to June 2008. The choice to deliver chemotherapy was based on biological prognostic factors. Anthracycline-based chemotherapy was administered in 83.5% patients.

## Background

Breast cancer is the first cause of tumour-related death in women in Italy [1-5]. Moreover, the incidence of breast cancer has been increasing over the last 15 years in industrialised countries as the result of both Rx-mammographic screening programs [1-5] and advances in breast cancer awareness. The increase in incidence due to mammography screening has been due to the increase in small T1 cancers [5,6]. In Italy, the pT1-pT1b incidence was 9.6% in 1988–1990 and 21.4% in 2005–2007 (AIRTUM, data not published; http://www.registri-tumori.it): these tumours were pN0 in 82%–85% of the cases.

The prognosis of these small cancers used to be considered very good, with a relapse-free survival rate at 10 years without adjuvant therapy of 90% [7-11]. However, some retrospective studies have reported a worse outcome, with 81%–86% of relapse-free survival at 10 years [12-15]. Furthermore, Fisher et al. [16] reporting survival data on 1,259 patients with pT1a-pT1b N0 breast cancer enrolled in 5 randomised NSABP trials, suggested that these patients could have benefited from adjuvant systemic therapy. Given that there is an ongoing debate on the adjuvant treatment of these tumours, we analysed the patients with pT1a-pT1b breast cancer enrolled in the NEMESI study, a retrospective observational study conducted in 2009 at 63 Italian oncological centres. We evaluated the adjuvant systemic treatments delivered, the variables which determined the choice to administer chemotherapy, the type of chemotherapy delivered and compliance with chemotherapy in pT1a-pT1b breast cancers.

## Methods

### Study design

NEMESI is a retrospective observational study conducted at 63 Italian oncological centres. The centres were extracted from a database of 319 oncological centres reported in the census of the Italian Association Medical Oncology (AIOM) [17] and stratified by geographical area (Northern, Central, Southern Italy, including Sicily and Sardinia) and kind of institution (public hospital, university hospital, research institute, private hospital, other), since these factors were considered to have an important impact on compliance with guidelines.

The endpoints of the NEMESI study were: to describe the adjuvant systemic treatments delivered in pathological stage I-II breast cancer, to evaluate the variables which determined the choice of adjuvant chemotherapy and the type of chemotherapy, to evaluate compliance with the treatment administered, and to compare the practice observed in Italian clinical settings with that proposed by international guidelines.

Criteria of eligibility were: women age ≥18 years; histological diagnosis of invasive breast cancer stage I-II (AJCC version VI) [18] who underwent surgery; at least one cycle of adjuvant chemotherapy and/or adjuvant hormonal therapy; availability of the following local staging and biological parameters: pT, pN, grading, estrogen receptor (ER), progesterone receptor (PgR), proliferative index (Ki67 or MIB-1), HER2. Candidates for adjuvant therapy with trastuzumab and/or radiotherapy on residual breast or thoracic wall and/or regional supraclavicular and/or internal breast lymph node stations were also eligible. Exclusion criteria were: neo-adjuvant chemotherapy and/or hormonal therapy, locally advanced and/or metastatic (stage III-IV) breast cancer, and in-situ carcinoma. Data were retrospectively retrieved by each site from the patients’ clinical records.

The protocol was reviewed by the independent ethics committee of the coordinating centre and notification of the study was sent to the ethics committees of each participating centre [19]. The protocol complied with the recommendations of the 18th World Health Congress (Helsinki, 1964) [20].

### Sample size determination and data collection

The study aimed at collecting data from the clinical records of not less than 1,300 and not more than 1,500 patients attending at least 50 oncologic centres. These figures correspond to 3.6% and 4.2% respectively of the new cases of early stage breast cancer recorded each year in Italy (about 40,000 cases, 90% of which are early stage), and 12.5% of the Italian oncologic sites (about 400 throughout Italy).

Each centre was requested to collect, within December 2009, the data of a minimum of ten and a maximum of 30 consecutive patients with early breast cancer between 1 January 2008 and 30 June 2008; with the only requirement being to collect the data of at least 33% of the patients undergoing adjuvant chemotherapy. This requirement was mandatory because the primary objective of the study was not to assess how many patients received adjuvant chemotherapy within that period but to identify the biological, staging and demographic parameters that determined: (i) whether adjuvant chemotherapy would be prescribed, and (ii) the type and schedule of chemotherapy selected. To ensure anonymity, the percentage of patients enrolled in the study could not exceed 50% of the patients attending the centre during the study period. According to the study protocol, central revision of all tissue samples was not done.

The data, collected on an electronic clinical report form, were submitted to automatic checks to assess completeness, correctness and internal coherence. Possible discrepancies or otherwise unreliable data were submitted to the investigator in the form of queries for clarification and/or resolution.

### Variables evaluated

In the NEMESI study patient and tumour characteristics, type of local-regional treatment and adjuvant systemic therapy, and compliance to chemotherapy were evaluated. In this NEMESI sub-study, only the pT1a-pT1b breast cancers were analysed. We evaluated: patient characteristics (age class, menopausal status), stage according to the TNM AJCC version VI classification, bio-pathological characteristics of the primitive tumour (histology, vascular invasion, grading, ER, PgR, HER2, and the proliferative index), type of surgery, adjuvant systemic therapy (chemotherapy, hormonal therapy, trastuzumab, concurrent adjuvant treatment with other experimental drugs), the variables associated with choice of administered adjuvant chemotherapy and compliance to chemotherapy. Moreover, in HER2-positive small tumours the variables associated with choice of administered adjuvant chemotherapy and trastuzamab were analysed.

### Statistical methods

In this survey 402 patients were examined with pT1a-pT1b breast tumour stage from a total of 1,894 patients enrolled in the NEMESI clinical study. The analysis was performed considering not only the reference population but also various subgroups decided in accordance with different analysis purposes. For most of these groups both descriptive analysis and inferential analysis were performed. As a descriptive analysis, continuous variables were summarised using descriptive statistics, including number of subjects: mean, standard deviation, and median, while for categorical variables summaries included counts of subjects and percentages. Pearson’s Chi-Square (*χ*2) test was performed in order to evaluate whether the frequency distribution of certain events observed in a sample (for example variables as age, menopausal status, grading, ER status) is consistent with their theoretical distributions. Chi-Square test for Specified Proportions was used to compare, in one way frequency tables, the homogeneity proportion between the general population (pathological stage I-II with the exclusion of pT1a, pT1b, considered as a reference distribution) and the distribution of the same variables in the subgroup of pT1a-pT1b patients. For both tests, the significance level used was equal to p = 0.05.

Multivariate logistic regression analysis was performed to assess the relationship between clinical and demographic variables and the type and treatment schedule of adjuvant chemotherapy administered with many covariates: age class, menopausal status, vascular invasion, ECOG performance status, type of surgery, TNM stage, ER, PgR, HER2 status, proliferative index. The logistic model contained only categorical variables. The continuous variables (e.g. proliferative index, ER, PgR, HER2) were categorized in different classes according to international indications.

The selection of variables to include in the model was defined using the stepwise procedure with a significant level of p = 0.05 to include variables in the model. In the logistic model the odds ratio estimates and their 95% confidence limits were also calculated. All statistical analysis was performed using SAS (Statistical Analysis System, SAS Institute Inc., Cary, NC, USA) version 9.1.3 for Windows.

## Results

In the NEMESI study 1,894 patients with pathological stage I-II breast cancer were enrolled by 63 Italian centres. Out of 1,894 cases included in this survey, 402 (21.2%) were pT1a-pT1b breast cancers. Patient and tumour characteristics are reported in Tables 1 and 2; no considerable relevant differences were observed between pT1a-pT1b breast cancers. Conservative breast surgery and node sentinel biopsy were performed in 86% and in 79.3% of pT1a-pT1b tumours, respectively (Table 1). The majority of the tumours were pN0 (78.8%) and showed G1-G2 grading (80.6%), absence of vascular invasion (67.5%), low proliferative index (71.8%), hormonal status positive (89.7%), HER2-status negative (88.12%) (Table 2). Patients and tumour characteristics of 402 pT1a-pT1b breast cancers were compared to 1,492 ≥pT1c tumours of NEMESI study (Table 3). The following variables were distributed in a statistically different manner between the groups: menopausal status (p = 0.015), conservative surgery (p <0.0001), grading (p < 0.0001), proliferative index (p < 0.0001), ER status (p = 0.02), hormonal receptor status (p = 0.006) and HER2 status (p = 0.009). Analysing the different categories of the statistically significant variables, it was observed that in 402 pT1a-pT1b breast cancers compared to other 1,492 cases, there were higher percentages of: postmenopausal patients (72.8% vs 68%; p = 0.05), tumours that underwent conservative surgery (86.7% vs 72.5%; p = 0.005) and sentinel node biopsies (79.4% vs 60.5%; p < 0.0001), tumours with grading G1 (25.1% vs 8.0%; p < 0.0001) and with a low proliferative index (70.6% vs 47.4%; p <0.0001). In the pT1a-pT1b tumours a lower incidence of prognostic biological factors associated with poor prognosis was observed: ER-negative status (12.7% vs 18.5%; p = 0.01), ER and PgR negative status (11.9% vs 17.9%; p = 0.01), and HER2-positive status (12.2% vs 18.7%; p = 0.006). These small breast cancers (≤ 1 cm) showed a higher percentage of pN0 (79.4% vs 56%; p <0.0001) compared to 1,492 ≥pT1c tumours of the NEMESI study.

**Table 1:** Patient characteristics and treatment

	**Total (pT1a and pT1b)**	**pT1a**	**pT1b**
**N. of patients**	402	82	320
**Distribution by age**			
18–34	7 (1.7%)	2 (2.4%)	5 (1.6%)
35–49	85 (21.1%)	19 (23.2%)	66 (20.6%)
50–69	230 (57.2%)	47 (57.3%)	183 (57.2%)
≥ 70	80 (19.9%)	14 (17.1%)	66 (20.6%)
**Menopausal status**			
Pre-	101 (25.1%)	25 (30.5%)	76 (23.8%)
Post-	293 (72.9%)	54 (65.9%)	239 (74.6%)
Missing	8 (2.0%)	3 (3.6%)	5 (1.6%)
**Surgery:**			
Breast conservative surgery	346 (86.0%)	65 (79.3%)	281 (87.8%)
Mastectomy	56 (13.9%)	17 (20.7%)	39 (12.2%)
**Node sentinel biopsy**			
Yes	319 (79.4%)	64 (78.0%)	255 (79.7%)
No	83 (20.6%)	18 (22.0%)	65 (20.3%)
**Axillary nodal dissection**			
Yes	137 (34.1%)	31 (37.8%)	106 (33.1%)
No	265 (65.9%)	51 (62.2%)	214 (66.9%)
**Radiotherapy**			
Yes	317/402 (78.9%)	58/82 (70.7%)	259/320 (81.0%)
No	85/402 (21.1%)	24/82 (29.3%)	61/320 (19.0%)

**Table 2:** Biopathological characteristics

	**Total (pT1a and pT1b)**	**pT1a**	**pT1b**
**N. of patients**	**402**	**82**	**320**
**Histology**			
Ductal	336 (83.6%)	70 (85.4%)	266 (83.1%)
Lobular	38 (9.5%)	7 (8.5%)	31 (9.7%)
Mixed	5 (1.2%)	0 (0.0%)	5 (1.6%)
Other	23 (5.7%)	5 (6.1%)	18 (5.6%)
**Vascular invasion**			
Yes	30 (7.5%)	3 (3.7%)	27 (8.4%)
No	267 (66.4%)	51 (62.2%)	216 (67.5%)
Unknown	105 (26.1%)	28 (34.1%)	77 (24.1%)
**Grading**			
G1	101 (25.1%)	18 (22.0%)	83 (25.9%)
G2	221 (55.0%)	46 (56.1%)	175 (54.7%)
G3	72 (17.9%)	15 (18.3%)	57 (17.8%)
Unknown	8 (2.0%)	3 (3.7%)	5 (1.6%)
**Proliferation index (Ki-67/MB1)**			
0–18%	283 (70.5%)	53 (64.6%)	230 (71.8%)
19–29%	52 (12.9%)	17 (20.7%)	35 (10.9%)
≥ 30%	54 (13.4%)	7 (8.5%)	47 (14.7%)
Unknown	13 (3.2%)	5 (6.1%)	8 (2.5%)
**ER status**			
ER positive (≥ 10%)	351 (87.3%)	65 (80.5%)	285 (89.1%)
ER negative (0–9%)	51 (12.7%)	16 (19.5%)	35 (10.9%)
**PgR status**			
PgR positive (≥ 10%)	300 (74.6%)	54 (65.9%)	246 (76.9%)
PgR negative (0–9%)	102 (25.4%)	28 (34.1%)	74 (23.1%)
Hormonal status*			
ER and/or PgR positive	354 (88.1%)	65 (81.7%)	287 (89.7%)
ER and PgR negative	48 (11.9%)	15 (18.3%)	33 (10.3%)
HER2 status**			
Positive	49 (12.2%)	19 (23.2%)	30 (9.3%)
Negative	344 (85.6%)	62 (75.6%)	282 (88.2%)
Unknown	9 (2.2%)	1 (1.2%)	8 (2.5%)
**pN status**			
pN0	319 (79.4%)	67 (81.7%)	252 (78.8%)
pN1 (1–3)	74 (18.4%)	12 (14.6%)	62 (19.4%)
pN2 (4–9)	6 (1.5%)	2 (2.4%)	4 (1.3%)
pN3 (≥ 10)	3 (0.7%)	1 (1.2%)	2 (0.6%)

* cut off: 10%

** HER2 status positive if: IHC 3+; or IHC 2+ and amplified by FISH,SISH,CISH; or amplified by FISH,SISH,CISH

**Table 3:** Patient and tumour characteristics of pT1a-pT1b tumours vs other stage I-II tumours in the NEMESI study

	**Pathological stage I-II (pT1a, pT1b excluded)**	**pT1a, pT1b**
**N. of patients**	1492	402
**Distribution by age**		
18–34	23 (1.5%)	7 (1.7%)
35–49	384 (25.7%)	85 (21.1%)
50–69	754 (50.5%)	230 (57.2%)
≥ 70	331 (22.2%)	80 (19.9%)
**Menopausal status**		
Pre-	465 (31.2%)	101 (25.1%)
Post-	1,014 (68.0%)	293 (72.8%)
Unknown	13 (0.9%)	8 (2.0%)
**Node sentinel biopsy**		
Yes	903 (60.5%)	319 (79.3%)
No	589 (39.5%)	83 (20.7%)
**Axillary nodal dissection**		
Yes	941 (63.1%)	137 (34.1%)
No	551 (36.9%)	265 (65.9%)
**Grading**		
G1	120 (8.0%)	101 (25.1%)
G2	741 (48.7%)	221 (55.0%)
G3	589 (39.5%)	72 (17.9%)
Unknown	42 (2.8%)	8 (2.0%)
**Proliferation index (Ki-67/MB1)**		
0–18%	707 (47.4%)	283 (70.6%)
19–29%	279 (18.7%)	52 (12.9%)
≥ 30%	461 (30.9%)	54 (13.4%)
Unknown	45 (3.0%)	13 (3.2%)
**ER status**		
ER positive (≥ 10%)	1,215 (81.4%)	351 (87.3%)
ER negative (0–9%)	276 (18.5%)	51 (12.7%)
Unknown	1 (0.1%)	0 (0.0%)
**PgR status**		
PgR positive (≥ 10%)	1,038 (69.6%)	300 (74.6%)
PgR negative (0–9%)	449 (30.1%)	101 (25.4%)
Unknown	5 (0.3%)	0 (0.0%)
Hormonal status*		
ER and/or PgR positive	1,228 (82.3%)	354 (88.1%)
ER and PgR negative	263 (17.6%)	48 (11.9%)
Unknown	1 (0.1%)	0 (0.0%)
HER2 status**		
Positive	279 (18.7%)	49 (12.2%)
Negative	1,185 (79.4%)	344 (85.6%)
Missing	28 (1.9%)	9 (2.2%)
**pN status**		
pN0	836 (56.0%)	319 (79.4%)
pN1 (1–3)	477 (32.0%)	74 (18.4%)
pN2 (4–9)	113 (7.6%)	6 (1.5%)
pN3 (≥ 10)	66 (4.4%)	3 (0.7%)

* cut off: 10%

** HER2 status positive if: IHC 3+; or IHC 2+ and amplified by FISH,SISH,CISH; or amplified by FISH,SISH,CISH

Adjuvant chemotherapy was delivered in 127 out of 402 patients (31.59%) with pT1a-pT1b breast cancer: in 27 of 82 pT1a tumours (32.9%) and in 100 of 320 pT1b tumours (31.25%). Patients and tumour characteristics were analysed to evaluate their influence on the decision to administer or not adjuvant chemotherapy (Table 4). Seventy-two out of 127 patients treated with chemotherapy were pN0 (56.69%). The multivariate logistic model analysis showed that younger age, grading G3, high proliferative index (≥ 30% Ki-67/Mib 1), ER negative status and HER2-positive status were significantly associated with the decision to administer adjuvant chemotherapy (Table 5).

**Table 4:** Adjuvant chemotherapy and patient/tumour characteristics

	**pT1a, pT1b total patients (N = 402)**	Patients treated with adjuvant chemotherapy (N = 127)*
**Distribution by age**		
18–34	7/402 (1.7%)	6/7 (85.7%)
35–49	85/402 (21.1%)	46/85 (54.1%)
50–69	230/402 (57.2%)	67/230 (29.1%)
≥ 70	80/402 (19.9%)	8/80 (10.0%)
**Menopausal status**		
Pre-	101/402 (25.1%)	52/101 (51.5%)
Post-	293/402 (72.8%)	72/293 (24.6%)
Unknown	8/402 (2.0%)	3/8 (37.5%)
**Grading**		
G1	101/402 (25.1%)	9/101 (8.9%)
G2	221/402 (55.0%)	66/221 (29.8%)
G3	72/402 (17.9%)	49/72 (68.0%)
Unknown	8/402 (2.0%)	3/8 (37.5%)
**Proliferation index (Ki-67/MB1)**		
0–18%	283/402 (70.6%)	55/283 (19.4%)
19–29%	52/402 (12.9%)	28/53 (52.8%)
≥ 30%	54/402 (13.4%)	41/54 (75.9%)
Unknown	13/402 (3.2%)	3/13 (23.0%)
**ER status**		
ER positive (≥ 10%)	351 /402(87.3%)	82/351(23.6%)
ER negative (0–9%)	51/402(12.7%)	45/51 (88.2%)
Hormonal status*		
ER and/or PgR positive	354 /402 (74.6%)	84/354 (24.0%)
ER and PgR negative	48/402 (25.4%)	43/48 (89.6%)
HER2 status**		
Positive	49/402 (12.2%)	36/49 (73.5%)
Negative	344/402 (85.6%)	90/344 (26.2%)
Missing	9/402 (2.2%)	1/9 (11.1%)
**pN status**		
pN0	319/402 (79.4%)	72/319 (22.6%)
pN1 (1–3)	74/402 (18.4%)	47/74 (63.5%)
pN2 (4–9)	6/402(1.5%)	5/6 (83.3%)
pN3 (≥ 10)	3/402 (0.7%)	3/3 (100%)

* cut off: 10%

** HER2 status positive if: IHC 3+; or IHC 2+ and amplified by FISH,SISH,CISH; or amplified by FISH,SISH,CISH

**Table 5:** Results of the multivariate logistic model analysis evaluating the probability of being treated with adjuvant chemotherapy for pT1a and pT1b breast cancers

**Variable**	**Odds ratio**	**95% CI**	**P value**
**Age range (years)**			0.0185
≥70	1		
18–34	NE	NE	
35–49	8.22	2.00-33.67	
50–70	2.44	0.69-8.58	
**Grading**			0.024
G1	1		
G2	3.48	0.56-5.01	
G3	8.33	1.82-33	
**ER receptor**			0.0007
Positive	1		
Negative	11.43	2.78-47.03	
**Proliferative index**			0.001
Low	1		
Medium	2.08	0.28-16	
High	9.10	2.57-33	
**HER2**			0.002
Negative	1		
Positive	9.09	2.73-33.3	

NE, not evaluable due to the small sample in this category

The multivariate logistic analysis confirmed that ER negative status, high proliferation index, and HER2-positive status were significantly associated with the decision to administer adjuvant chemotherapy in patients with pT1b (Table 6), while it could not be performed for pT1a due to the low number of patients (=27). Both analyses excluded the evaluation of axillary lymph node status from the model fit because the distribution of population was unbalanced and it was impossible to estimate the values for the different categories.

**Table 6:** Results of the multivariate logistic model analysis evaluating the probability of being treated with adjuvant chemotherapy for pT1b breast cancers

**Variable**	**Odds ratio**	**95% CI**	**P value**
**ER receptor**			0.001
Positive	1		
Negative	21.30	3.40-133.28	
**Proliferative index**			<0.0001
Low	1		
Medium	15	0.86-21.31	
High	20	5.56-100	
**HER2**			<0.0001
Negative	1		
Positive	20	4.35-100	

We analysed the types of adjuvant chemotherapy administered in 127 patients with pT1a-pT1b tumours. Anthracycline without taxane regimen was administered in 59.1% of patients, anthracycline with taxane in 24.4%, CMF-like in 14.2% and taxane without anthracycline in only 2.4% (Table 7).

**Table 7:** Type of chemotherapy administered in 127 patients with pT1a, pT1b breast cancers

	**All pts (N = 127)**	**pT1a pts (=27)**	**pT1b pts (N = 100)**
**Regimen**			
CMF-like	18/127 (14.2%)	3/27 (11.1%)	15/100 (15%)
Anthracycline without taxane	75/127 (59.1%)	21/27 (77.8%)	54/100 (54%)
Anthracycline with taxane	31/127 (24.4%)	3/27 (11.1%)	28/100 (28%)
Taxane without anthracycline	3/127 (2.4%)	0	3/100 (3%)

The small number of patients in the groups treated with different adjuvant chemotherapy regimens did not allow for a statistical evaluation of the patient and tumour characteristics which could have influenced the choice of adjuvant chemotherapy type.

In the few older patients (≥ 70 years) treated with chemotherapy, a CMF-like regimen was administered in 50%; in none of these patients was anthracycline and taxane-based adjuvant therapy chosen. A CMF-like regimen was chosen more frequently in postmenopausal patients (18.1% vs 9.6%), in patients with grade G1 tumours (22.2% vs 12.2% G3) and in patients with cancers having low or moderate proliferative indexes (14.5% and 25% respectively, versus 4.9% high proliferative index). In pT1b tumours, anthracycline and taxane-based regimen was chosen more frequently than in pT1a breast cancers (28% in pT1b vs 11.1% in pT1a) (Table 7).

Regarding compliance to adjuvant chemotherapy, it was observed that 105 of 127 patients (82.67%) received 4–6 cycles. Delays (> 7 days versus planned), were reported in 20 patients (15.7%), with a median delay of 7 days (range 7–28). The definitive interruption of chemotherapy occurred in only eight patients: three due to toxicity or severe adverse event, four due to the patient’s decision and one due to the investigator’s decision.

Hormonal therapy, planned in 351 of the 354 patients with hormonal receptor-positive tumours, was administered in 346 patients (97.7%). In pT1a-pT1b breast cancers, tamoxifen with or without LHRH was the hormonal therapy more frequently administered (85.3%) in premenopausal patients; aromatase inhibitors for 5 years (67.3%) was the endocrine treatment more often utilised in the postmenopausal setting, where tamoxifen for 2–3 years followed by aromatase inhibitor for 3–2 years was administered in 17.1% of patients (Table 8).

**Table 8:** Type of hormonal therapy administered according to menopausal status in 346 pT1a and pT1b tumours

	**Premenopausal (N = 89)**	**Postmenopausal (N = 257)**
**Hormonal therapy**		
Tamoxifen 5 y	11 (12.3%)	35 (13.6%)
Tamoxifen 5 y + LHRH	65 (73.0%)	0 (0.0%)
Tamoxifen 2-3y→Aromatase Inhibitor 3–2 y	3 (3.3%)	44 (17.1%)
Aromatase Inhibitor 5 y	1 (1.1%)	173 (67.3%)
Aromatase Inhibitor 5 y + LHRH	6 (6.7%)	1 (0.4%)
LHRH alone	1 (1.1%)	0 (0.0%)
Other	2 (2.2%)	4 (1.6%)

### Adjuvant chemotherapy in triple-negative pT1a and pT1b tumours

Triple-negative (ER = 0%, PgR = 0%, HER2-negative) pT1a-pT1 b tumours were 26 (6.5%); 2 were pT1a and 24 were pT1b. Adjuvant chemotherapy was administered in 88.4% of patients (23/26): in 1 of 2 patients with pT1a (50%) and in 22 of 24 pT1b patients (91.66%). A CMF-like regimen was administered in 6 patients (26.1%), anthracycline without taxane in 11 (47.8%), anthracycline with taxane in 5 (21.7%) and taxane without anthracycline in 1 (4.3%).

### Adjuvant chemotherapy and adjuvant trastuzumab in pT1a and pT1b HER2-positive breast cancers

Forty-nine pT1a-pT1b tumours were HER2-positive (12.1%). Adjuvant chemotherapy was delivered to 36 of these 49 patients (73.46%): in 13/19 pT1a (68.4%) and in 23/130 pT1b (76.6%). Patient and tumour characteristics are reported in Table 9. Adjuvant chemotherapy was administered in 100% (19/19) of the younger patients (18–49 years) and in 100% (6/6) of patients with metastatic lymph nodes, in 90% (18/20) of tumours with negative hormonal receptors, and 69.6% and 84.2% of G2 and G3 tumours, respectively.

**Table 9:** Adjuvant chemotherapy and adjuvant trastuzumab by tumour and patient characteristics in HER2-positive pT1a and pT1b breast cancers (n = 49)

	**All HER2-positive tumours**	HER2-positive tumours treated with adjuvant chemotherapy*	HER2-positive tumours treated with adjuvant chemotherapy and trastuzumab *
No. of patients	**49**	**36 (73.5%)**	**30 (61.2%)**
**Menopausal status**			
pre-	19	18 (94.7%)	14 (73.7%)
post-	30	18 (60.0%)	16 (53.3%)
**Age (years)**			
18–34	2	2 (100.0%)	2 (100.0%)
35–49	17	17 (100.0%)	12 (70.6%)
50–69	25	16 (64.0%)	15 (60.0%)
≥ 70	5	1 (20.0%)	1 (20.0%)
**Hormonal receptor status**			
positive	29	18 (62.1%)	15 (51.7%)
negative	20	18 (90.0%)	15 (75.0%)
**LN status**			
pN0	43	30 (69.7%)	24 (55.8%)
pN1	4	4 (100.0%)	4 (100.0%)
pN2	2	2 (100.0%)	2 (100.0%)
**Grading**			
G1	2	1 (50.0%)	0 (0.0%)
G2	23	16 (69.6%)	13 (56.5%)
G3	19	16 (84.2%)	16 (84.2%)

* Values and percentages are based on the all HER2-positive patients

We observed that 30 out of 36 patients treated with adjuvant chemotherapy were pN0 and in these patients the choice of administered chemotherapy was independent of other patient and tumour characteristics and based only on HER2-positivity. An anthracycline-based regimen was administered in 34 of 36 HER2-positive patients (94.4%) (anthracycline without taxane in 72.2% and anthracycline with taxane in 22.2%); a CMF-like regimen in 1 patient (2.7%) and taxane without anthracycline in 1 patient (2.7%). In 30 out of 36 patients treated with chemotherapy, also adjuvant trastuzumab was administered. Trastuzumab was delivered in all six patients with lymph node involvement and in 24 patients with pN0 disease (Table 9).

## Discussion

In the NEMESI study, a retrospective study which enrolled 1,894 pathological stage I-II breast cancers, 402 pT1a-pT1b tumours were included. A multivariate analysis conducted to evaluate the influence of patient and tumour characteristics on the decision to administer or not adjuvant chemotherapy in pT1a-pT1b breast cancers, showed that younger age, grading G3, high proliferative index, ER-negative status and HER2-positive status were significantly associated with the decision to administer adjuvant chemotherapy. In the patients treated with adjuvant chemotherapy, an anthracycline-based regimen was administered in 83.5% (anthracycline without taxane in 59.1% and anthracycline with taxane in 24.4%) while a CMF-like regimen was administered in only 13.38%. In our study compliance to adjuvant chemotherapy was high (82.67% of patients received 4–6 planned cycles) and the definitive interruption of chemotherapy occurred in only eight patients.

The adjuvant systemic therapy of small tumour size with no axillary lymph node involvement is controversial. The risk of relapse is related to stage of tumour (tumour size and lymph node status) and to biological characteristics. Therefore, in pT1a-pT1b tumours, which are pN0 in 82%–85% of the cases, the biological markers are utilised during treatment decision-making. In our study, the variables significantly associated with the decision to administer adjuvant chemotherapy in pT1a-pT1b breast cancers were younger age and the biological markers associated with a poor prognosis (grading G3, high proliferative index, ER-negative status and HER2-positive status) [14,16,21-28], and also predictive of chemoresponsivity in the neoadjuvant setting.

Several changes in indication to adjuvant systemic therapy occurred for patients with node-negative tumours ≤ 1 cm in size according to 1998–2007 St Gallen Consensus Conference guidelines. In the 1998 St Gallen Consensus Conference the population with <10% of relapse was not considered for adjuvant systemic therapy [29]. The 2005 Consensus Conference made a fundamental change in the algorithm for the selection of adjuvant systemic therapy for early breast cancer, considering first endocrine responsiveness and then the risk of relapse. The risk allocation of tumours below 1 cm in size and negative nodes remained still controversial [30]. The 2007 St Gallen Consensus Conference [31] utilised the biological factors associated to worse prognosis, considered singularly or together, to identify the endocrine non responsive tumours suitable for only adjuvant chemotherapy, and to identify the incompletely or highly endocrine responsive tumours suitable, according to risk of relapse, for addition of adjuvant chemotherapy to hormonal therapy, irrespective of tumour size. However, some but not all panel members viewed pT≤1 cm tumours with node-negative disease as representing low risk even if higher grade and/or younger age. The NCCN Practice Guidelines 2007 http://www.nccn.org recommended adjuvant chemotherapy only in tumours between 6 mm and 10 mm without metastases in lymph nodes (pT1b pN0): in ER-negative pT1b pN0 (both HER2-negative and HER2-positive) and, in addition to hormonal therapy, in ER positive pT1b pN0 moderate/poorly differentiated or with unfavourable features (both HER2-negative and HER2-positive).

We report that an anthracycline-based regimen was administered in 83.5% of patients (anthracycline without taxane in 59.1% and anthracycline with taxane in 24.4%) while CMF-like anthracycline-regimens (without or with taxane) highlights that if the decision was to administer chemotherapy, the most active regimen was selected, also in small breast cancers. This trend was observed also in all patients with stage I-II breast cancer enrolled in the NEMESI study [32] as well as in the NEMESI subgroup of triple-negative tumours [33].

Our study has some limits. Although the majority of pT1a-pT1b breast cancers had favourable prognostic factors, as reported in other retrospective studies [7-11], adjuvant chemotherapy was delivered in 31.59% of patients. This percentage is considerable but may not reflect the clinical practice and must be evaluated considering both the eligibility criteria of NEMESI (the patients enrolled must have received at least one cycle of adjuvant chemotherapy and/or adjuvant hormonal therapy) and the requirement that each centre had to collect the data of at least 33% of the patients undergoing adjuvant chemotherapy. It is necessary consider this limit also when we reported that 36 out of 49 patients with HER2-positive small breast cancer were treated with adjuvant chemotherapy (73.46%). Thirty out of these 36 HER2-positive patients were pN0 and in these patients the choice of administered chemotherapy was independent of other patient and tumour characteristics and based only on HER2-positivity, considered a poor prognostic factor [22-24], as well confirmed by recent studies [25-28]. Adjuvant trastuzumab was administered in 30 of 36 patients who received chemotherapy. HER2-positivity is a predictive factor of trastuzumab response, but although five out of six randomised phase III trials reported marked benefit of adjuvant trastuzumab for disease-free and overall survival (with reduction of recurrence and mortality by 20-40%) [34-38], there are no data on trastuzumab in pT1a-pT1b HER2-positive breast cancer. On the other hand, there is indirect evidence. In the BCIRG006 and HERA subgroup analyses adjuvant trastuzumab did not result in different rates of risk reduction among HER2-positive breast cancers in function of nodal status or tumour size [35,39,40]. More data supporting the use of adjuvant trastuzumab in small node-negative HER2-positive breast cancer emerged from three recently reported retrospective investigations. In a French multicenter series from 2002 to 2008, 97 patients with pT1a,b pN0 HER2-positive tumours were identified. Forty-one patients (42%) had been treated with adjuvant trastuzumab-based therapy with (n = 38) or without (n = 3) chemotherapy [41]. The decision to administer adjuvant trastuzumab was significantly associated with a negative hormonal receptor status, a high Eltson-Ellis grade, a moderate/high mitotic index, and the date of the diagnosis (before or after the HERA results were released). With a median follow-up of 29 months, there were no recurrences in patients treated with trastuzumab-based therapy while 5 of 56 patients who did not receive trastuzumab had developed a recurrence. Another single-institution retrospective study included 485 women with node-negative, HER2-positive tumours ≤2 cm treated in the pre- (2002–2004) and post- (2005–2008) trastuzumab era [42]. Events of disease recurrence were more frequent in the pre-trastuzumab group as compared with the post-trastuzumab group. A third study reported the breast cancer specific 5-year survival of HER2-positive pT1a and pT1b pN0 breast cancer in 20,188 patients identified in the California Cancer Registry [43]. It was significantly shorter among HER2-positive breast cancer compared to HER2-negative patients (p = 0.0001) in the 2000–2004 era, while there was no difference in the 2005–2007 era, after the introduction in clinical practice of adjuvant trastuzumab.

The 2007 St Gallen Consensus Conference did not recommend adjuvant trastuzumab in women with a primary tumour < 1 cm in size and with no axillary node involvement [31], and also the 2007 NCCN Guidelines did not indicate trastuzumab in tumours <1 cm. On the other hand, the more recent version of the NCCN Guidelines 2011 (v.2.2011) recommend the use of adjuvant trastuzumab in women with node-negative tumours (both HR-positive and HR-negative) that are 0.6 to 1.0 cm as category 2A recommendation, because patients with tumours 1 cm or smaller and node negative were not consistently included in the available clinical trials. The majority of the Panel members of the 2011 St Gallen Consensus Conference were willing to extended adjuvant trastuzumab to patients with pT1b, but not pT1a pN0 disease [44].

Moreover, hormonal therapy, planned in 351 patients out of 354 hormonal receptor-positive pT1a-pT1b tumours, was administered in 346 patients (97.7%). These data are very different from those reported by an audit of clinical practice in Italy conducted in March 2000 regarding adjuvant systemic therapies prescribed for breast cancer. In this audit it resulted that endocrine therapy was not prescribed in 102 out of 541 patients (19%) with endocrine-responsive disease [45].

## Conclusions

In conclusion, the choice to deliver adjuvant chemotherapy to patients with pT1a-pT1b breast cancer treated at 63 Italian oncological centres from January 2008 to June 2008 was based on tumour biology. When it was decided to administer adjuvant chemotherapy, the most active regimens, anthracycline-based, were selected. Compliance to treatment was excellent.

## Competing interests

The authors declare that they have no competing interests except for D. Dondi who is an employee of Sanofi-Aventis.

## Authors’ contribution

All authors have contributed to the conception and design of the study, acquisition and interpretation of data. Stefania Gori was responsible for drafting the manuscript. All authors have given approval to this version of the manuscript.

## Pre-publication history

The pre-publication history for this paper can be accessed here:

http://www.biomedcentral.com/1471-2407/12/158/prepub
